# Concealed, Unobtrusive Ear-Centered EEG Acquisition: cEEGrids for Transparent EEG

**DOI:** 10.3389/fnhum.2017.00163

**Published:** 2017-04-07

**Authors:** Martin G. Bleichner, Stefan Debener

**Affiliations:** ^1^Neuropsychology Lab, Department of Psychology, European Medical School, University of OldenburgOldenburg, Germany; ^2^Cluster of Excellence Hearing4all, University of OldenburgOldenburg, Germany; ^3^Center for Neurosensory Science and Systems, University of OldenburgOldenburg, Germany

**Keywords:** mobile EEG, ear-centered EEG, ear EEG, transparent EEG, wearable EEG

## Abstract

Electroencephalography (EEG) is an important clinical tool and frequently used to study the brain-behavior relationship in humans noninvasively. Traditionally, EEG signals are recorded by positioning electrodes on the scalp and keeping them in place with glue, rubber bands, or elastic caps. This setup provides good coverage of the head, but is impractical for EEG acquisition in natural daily-life situations. Here, we propose the transparent EEG concept. Transparent EEG aims for motion tolerant, highly portable, unobtrusive, and near invisible data acquisition with minimum disturbance of a user's daily activities. In recent years several ear-centered EEG solutions that are compatible with the transparent EEG concept have been presented. We discuss work showing that miniature electrodes placed in and around the human ear are a feasible solution, as they are sensitive enough to pick up electrical signals stemming from various brain and non-brain sources. We also describe the cEEGrid flex-printed sensor array, which enables unobtrusive multi-channel EEG acquisition from around the ear. In a number of validation studies we found that the cEEGrid enables the recording of meaningful continuous EEG, event-related potentials and neural oscillations. Here, we explain the rationale underlying the cEEGrid ear-EEG solution, present possible use cases and identify open issues that need to be solved on the way toward transparent EEG.

## Introduction

Electroencephalography (EEG) is a well-established noninvasive method to record the electrical activity of the human brain using electrodes placed on the scalp. EEG is extensively used for clinical applications such as epilepsy diagnostics (Noachtar and Rémi, 2009), sleep staging (Campbell, 2009), diagnosis of hearing loss (Paulraj et al., 2015), anesthesia monitoring (Marchant et al., 2014), and brain-computer interfaces (Shih et al., 2012). Moreover, EEG serves as a fundamental research tool for understanding human brain function (Lopes da Silva, 2013). EEG signals are known for their high temporal resolution allowing to observe changes in neural activity at millisecond precision. Compared to neuroimaging techniques such as magnetic resonance imaging (MRI) or magnetoencephalography (MEG), EEG hardware is available at very low cost, relatively easy to use, and very flexible in its application. For instance, clinically useful EEG signals can already be acquired with as few as three electrodes (e.g., Jewett and Williston, 1971), but current clinical and research practice is the simultaneous acquisition from multiple scalp electrodes. The use of 20, 100, or even 200 electrodes is common and the resulting recordings are rich in spatial detail. However, multi-channel EEG is feasible only if a net or cap is used to keep sensors in place. Hence, the established EEG sensor and cap systems are bulky. They often come with loose wires, are clearly visible, and may not provide good signal quality over prolonged periods of time; and if they do, they are not comfortable to wear. Here, we discuss recently developed alternatives to conventional EEG acquisition technology, with a particular focus on solutions that aim for daily-life application. We introduce the concept of transparent EEG as a new approach to acquire electrophysiological data with minimal inconvenience for the person that is monitored and show how this can be implemented using ear-EEG.

The term mobile EEG has been used to describe the study of EEG-derived brain signals during motion (De Vos et al., 2014; Gramann, 2014). The role of the motor system has been recognized in cognitive neuroscience research: motor cognition, for instance, states that cognitive processing is embedded into actions and that the motor system contributes to cognitive processing (Jeannerod, 2008). Cognitive processes are different between rest and movement conditions (e.g., vision for action vs. vision for recognition, Goodale et al., 1991, or the interference of cognitive effort and gait stability, Al-Yahya et al., 2011) and the motor system even closely interacts with sensory processing (Schafer and Marcus, 1973). Accordingly the ecological validity of cognitive neuroscience research depends to a significant degree on the ability of studying the brain during natural motion (Ladouce et al., 2017). Even subtle motion, however, may distort signal quality. This drawback resulted in lab procedures that aim for highly artificial, motion-minimized recording situations. Yet, all behavior including speech is expressed as motion of (parts) of the human body. Thus, to study cognition during motion the availability of technology that tolerates motion appears advantageous. In this regard, EEG has a clear advantage over MRI or MEG since it can be made portable and has a higher motion tolerance. Several developments such as active or shielded electrodes (Metting van Rijn et al., 1990) and new sensor technology (Cömert and Hyttinen, 2015; Goverdovsky et al., 2015) may increase the degree of motion-tolerance even further. Here, we refer to mobile EEG as a technology that does not require the user to remain still. Mobile EEG systems are supposed to tolerate at least a modest degree of motion during signal acquisition, such as free walking at leisure pace (e.g., Debener et al., 2012). Not all mobile EEG solutions that tolerate user motion are mobile in the sense that they can easily be re-located. Indeed, mobile EEG research often combines EEG acquisition with motion-tracking and other recording modalities (Ojeda et al., 2014), which results in highly complex but stationary recording set-ups. This means that setups for mobile EEG are not necessarily portable.

More recently developed EEG amplifier-sensor systems are small enough to fit into a trouser pocket. We refer to these small EEG systems that can easily be carried around as portable EEG. Interestingly, some portable EEG systems do not even require a computer, as recordings can be stored on the device or transmitted wirelessly to a smartphone (e.g., Stopczynski et al., 2014a; Debener et al., 2015). It is important to note that portable EEG devices are not necessarily motion-tolerant. It remains to be seen whether recently developed portable EEG systems feature good signal quality during gross body motion.

Most conventional EEG systems require skin abrasion and application of an electrolyte paste to achieve a low-impedance skin-electrode contact. The preparation of multi-channel EEG recordings and the cleanup afterwards is hence time consuming for both experimenter and participant. Typically hair washing is required after completion of an EEG recording session. To overcome these drawbacks, dry EEG sensor solutions have been developed. Some of these systems are now market-ready and do indeed require very little preparation time and no hair-washing afterwards (Zander et al., 2011; Fiedler et al., 2014). However, signal quality issues remain (Bertrand et al., 2013; Tautan et al., 2014; Mathewson et al., 2017), and the high-impedance skin-electrode contact of dry sensors may reduce motion-tolerance to a level below that of traditional wet EEG technology. Moreover, dry EEG systems require a constant pressure of electrodes onto the skin, and this pressure bears the risk of increasing discomfort and headaches. In order to avoid subsequent hair washing, electrolyte fluids as skin-contact elements have been developed (e.g., Alba et al., 2010). Several popular consumer EEG solutions are based on a similar idea. By using dry electrode caps, or by combining electrolyte fluids with a headset, a self-fitting of EEG sensors becomes feasible.

This self-fitting characteristic appears important for several of the use cases presented below, and may be best described with the term wearable EEG. However, as mentioned above, wearable consumer-EEG systems, while portable, are not necessarily motion-tolerant. In 2012, we therefore introduced a consumer-EEG conversion kit and showed that low cost, wireless EEG can produce good EEG signal quality while walking outdoors—if combined with conventional cap EEG electrodes (Debener et al., 2012).

Mobile EEG, portable EEG, and wearable EEG labels are not consistently used in the literature, and our short characterizations may not apply to all published uses of these terms. For daily-life applications, however, these and further characteristics have to be combined into a single, next generation EEG approach.

We call this new approach transparent EEG. A transparent EEG is defined as a portable, motion-tolerant, self-applicable, highly unobtrusive, near invisible, and comfortable to wear EEG system. These requirements hold equally for sensor and amplifier technology. A transparent EEG should consist of sensors that are very small, near invisible and maintain good contact with the skin, preferably over many hours. All wires should be bundled and connect to an amplifier unit located in close proximity to the sensor array, to avoid long wires and minimize the risk of interference (Simakov and Webster, 2010). Accordingly, the amplifier must be head-mounted and should therefore be small enough in size to fit into glasses, behind the ear, or into in-ear devices similar to a modern hearing-aid. An amplifier used for transparent EEG should have low power consumption (or be easily and quickly rechargeable) and transmit signals wirelessly to a recording and signal-processing unit.

Modern smartphone technology features wireless communication and sufficient on-board storage and computational power to support EEG acquisition and basic signal processing (Stopczynski et al., 2014b). Importantly, such a system would allow for undisturbed natural social interactions and natural daily activities, and should not be more of a hassle to use than a pair of glasses, a hearing aid or a smartwatch.

In summary, a transparent EEG is a convenient EEG solution that allows to record brain signals relevant for a particular application with minimal disturbance to the users, short setup time, and long recording times. There is no exclusive way on how to implement a transparent EEG, but hardware miniaturization and convenient and unobtrusive sensor placement are critical. Consequently, transparent EEG has the potential to extend the usage of EEG to a wide variety of applications and situations that are not easily accessible with classical EEG solutions. In the remainder of this manuscript we argue that ear-EEG is one promising approach to implement transparent EEG and discuss our ear-EEG sensor approach using cEEGrid technology.

Aiming toward transparent EEG, it has been found that good signal quality and wearing comfort can be achieved with miniaturized EEG electrodes (Nikulin et al., 2010). Likewise, a number of ear-EEG systems featuring miniaturized EEG sensors have been proposed in recent years (Looney et al., 2012, 2014; Kidmose et al., 2013; Lee et al., 2014; Bleichner et al., 2015; Debener et al., 2015; Norton et al., 2015). Looney et al. (2012) pioneered modern EEG acquisition by placing electrodes into the outer ear canal and the concha. While replicating their work (Bleichner et al., 2015), we noticed that the resulting amplitudes are much smaller than what can be expected from scalp-EEG, which is due to the small distance between electrodes. As described below, it is hence worthwhile to consider locations around the ear as an intermediary between scalp-EEG and in-ear EEG. For this article we define ear-EEG systems as devices that place all necessary EEG sensors (i.e., recording electrode, ground and reference) in the outer ear canal, the concha, or the area around the ear.

By directly comparing simultaneously acquired scalp-EEG and ear-EEG signals, several independent laboratories have shown that ear-EEG can capture brain signals that are closely related to those recorded with scalp-EEG (Mikkelsen et al., 2015; Mirkovic et al., 2015; Bleichner et al., 2016; Zibrandtsen et al., 2016). In contrast to the classical EEG cap, ear-EEG sensors can be worn comfortably and are not more noticeable than hearing-aids or (in-ear) headphones. Ear-EEG sensors interfere much less with a participant's normal behavior and can be worn for many hours while maintaining good signal quality (Debener et al., 2015). First studies combining portable EEG systems with ear-EEG sensor technology and wireless smartphone-acquisition (Debener et al., 2015) have already been conducted, but commercially available portable EEG amplifier solutions are still too bulky to fit behind the ear. However, it is foreseeable that this will change in the near future (Zhang et al., 2013). These developments show that transparent EEG is well within reach. In the following section, we will present our ear-EEG approach in more detail.

## Ear-EEG with cEEGrids

**Figure 1:**
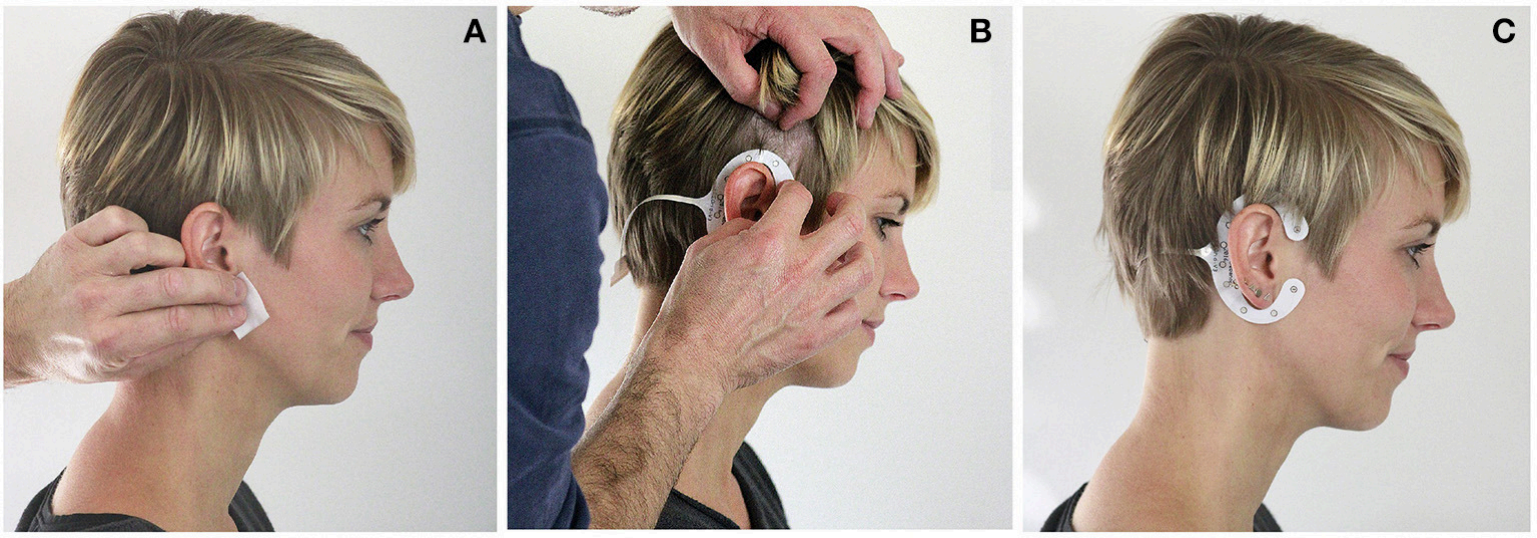
**cEEGrid application. (A)** The skin around the ear is cleansed with alcohol swabs. **(B)** The hair around the ear is pushed aside and the cEEGrid is placed around the ear with an adhesive tape. Good electrode skin conductance is assured by a drop of electrode gel on each electrode. **(C)** The applied grid allows for stable EEG recordings over several hours.

Based on the results of Bleichner et al. (2015), which showed that an in-concha electrode referenced to an above the ear electrode was sensitive to a P300 event-related potential (ERP), we have developed the cEEGrid (Debener et al., 2015). Unlike most other EEG electrodes, cEEGrid sensor arrays are printed, using flex-print technology (see www.ceegrid.com, for further details). cEEGrids are placed around the ear and hold firmly on the skin with an adhesive (Figure 1).

The flexprint material includes several layers of a biocompatible polyamide. All conductive parts of the currently available cEEGrid (3rd generation) are made from conductive Ag/AgCl based polymer thick film ink. The conductive surface of the contacts is circular with a diameter of 3 mm, and the distance between electrodes located within a cEEGrid is either 12 or 18 mm (center to center). The number of electrodes (10) as well as the size and shape of the cEEGrid were inspired by pilot recordings and previous experience with around the ear multi-channel EEG recordings using miniaturized sintered Ag/AgCl electrodes (Bleichner et al., 2015). The 10 electrodes per grid are arranged in a c-shape and are attached around the ear using a double-sided adhesive tape. The use of a small amount of electrolyte enables low impedance electrode-skin contact. Importantly, cEEGrid sensors do not dry out quickly, as the electrode skin connection is sealed by the adhesive tape around the electrodes. This solution comes with high wearing comfort and enables stable skin-electrode impedances over many hours.

Prior to electrode placement the skin is cleansed with an alcohol tissue. Afterwards the protective cover of the adhesive tape is removed and the cEEGrid is attached around the ear (Figure 1). The exact positioning of the cEEGrid depends on the shape of the user's ear, and hence varies slightly between participants and ears. It should be avoided that the cEEGrids touch the auricle, as this can result in discomfort after some time. If the cEEGrids do not touch the ear they are tolerated very well, even when worn over extended periods of time (>7 h, Debener et al., 2015).

**Figure 2:**
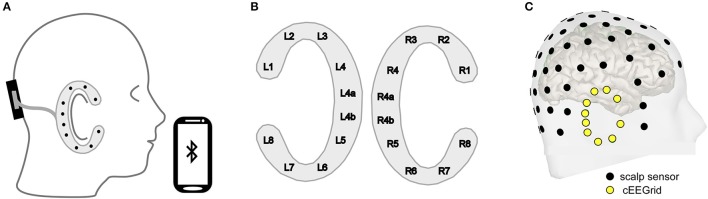
**(A)** A miniature, wireless EEG amplifier (black; https://mbraintrain.com/) is attached to the back of the head with a headband, and the cEEGrid is connected with the amplifier. The signal is transmitted wirelessly to a smartphone via Bluetooth. Android smartphones can be used for signal acquisition and stimulus presentation. **(B)** The cEEGrid electrodes are arranged in a C-shape. R4a and R4b may be used as ground (i.e., driven right leg) and reference electrodes. **(C)** Digitized electrode positions for a representative individual illustrating high-density equidistant EEG cap (black) and cEEGrid locations (yellow). The visualization was generated with the Brainstorm 3 software.

In our lab we generally combine cEEGrids with a small, wireless EEG amplifier (Smarting; www.mbraintrain.com), which is placed at the back of the head using a head band (Figure 2A). The headband can also serve to keep the upper part of the cEEGrid attached to the head. The cEEGrids are designed as disposables but can be reused if handled with care. The two electrodes located directly behind the right ear (R4a and R4b, see Figure 2B) serve as common and reference electrodes in our setup. This leaves eight electrodes distributed around the ear on the reference side and another 10 electrodes if a second cEEGrid is applied on the other side of the head.

## cEEGrid sensitivity—modeling

Due to effects of volume conduction an EEG signal that is recorded at a particular scalp location may be best regarded as reflecting a mixture of several sources that may be located close, or further away from a particular recording channel. Every single EEG channel, i.e., a recording electrode relative to a reference electrode, may be regarded as a spatial filter (Nunez and Srinivasan, 2009). Whether a particular EEG channel is sensitive to a particular neural source depends (among other aspects) on the distance between the source and the electrode, the orientation of the source relative to the electrodes, and the relative distance of the recording electrodes to each other. While superficial strong sources of radial orientation may be picked up by nearby electrodes, tangential sources may contribute stronger to more distant than nearby electrodes (Väisänen et al., 2008).

**Figure 3:**
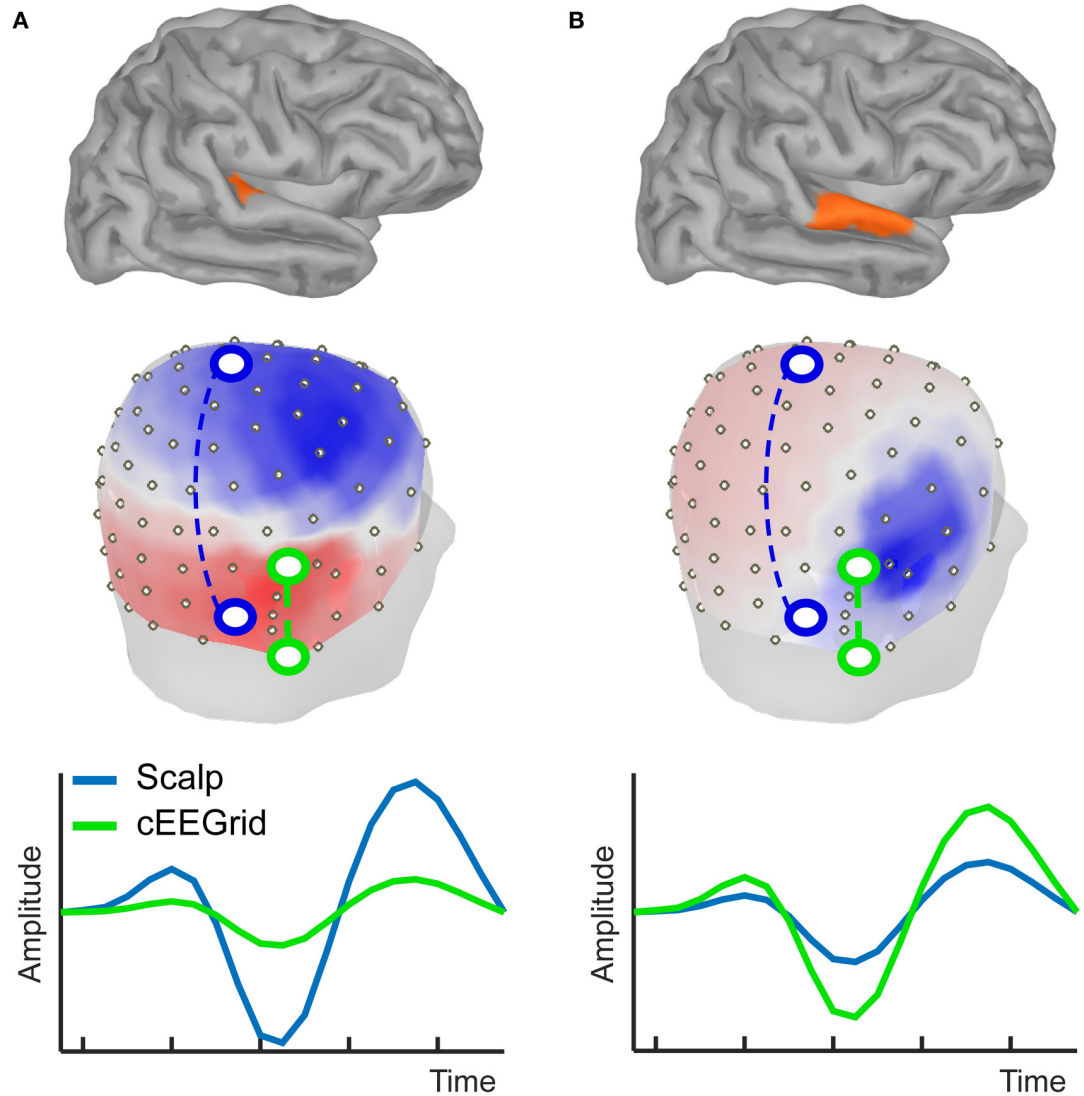
**Simulation of forward projections of two different cortical sources, one in transverse temporal cortex (A)** and the other one in superior temporal cortex **(B)**. Forward projections to the scalp show clear topographical differences, as illustrated by the voltage maps. Different example bipolar channels as indicated in green and blue on the 3D head model show differential sensitivity to the two sources. Source amplitudes are shown in arbitrary units. The line plots are scaled to maximum activity for each source.

The cEEGrid is placed on the hair free skin around the ear including the mastoid bone and is therefore partly located over the inferior temporal cortex (Figure 2C). If distance between source and electrode alone mattered, the cEEGrid should be most sensitive to signals that originate from the temporal lobe. However, source orientation matters as well, as we demonstrate here in a simulation (Figure 3) that uses a forward model (Tadel et al., 2011). This shows how source orientation influences its projection onto the scalp, and hence how well it is “seen” by a cap-EEG or ear-EEG channel. Specifically, we defined two nearby cortical regions in the right temporal lobe, which differ in orientation relative to the plane of the ear; the transverse temporal cortex (Figure 3A) and part of the superior temporal cortex (Figure 3B). Activation values for all vertices but the ones in the respective source region were set to zero, whereas for the vertices in a given source region we assumed a biphasic cortical activation pattern. EEG electrode positions used were based on two previous studies (Bleichner et al., 2016; Mirkovic et al., 2016), comprising 84 scalp channels arranged in an equidistant cap and 18 channels from two cEEGrids.

Figure 3 shows that the neural activation in the transverse temporal cortex projects primarily to the top of the head, with a polarity reversal between the upper and lower part of the head (Figure 3A). Consequently, the largest potential difference can be recorded with a bipolar channel consisting of an electrode on the side of the head (e.g., mastoid) and on the top of the head (e.g., vertex). Hence, it is well captured with a classical EEG cap. However, the source projects rather uniformly to those locations covered by the cEEGrid. Hence, the signal captured even with the most optimally arranged bipolar cEEGrid channel will be small in amplitude. The second source was placed on the superior temporal gyrus, and, despite close proximity to the first source, the near radial projection gives rise to a very different scalp distribution. Using cEEGrid electrodes above and below the ear this source can be recorded well. The vertex-mastoid cap-EEG channel on the other hand captures a relatively small signal.

This artificial, highly simplified example shows that scalp locations around the ear are suited to capture brain activity. Whether or not the signal of interest can be captured with electrodes around the ear depends on the exact position and orientation of the corresponding source(s). The advantage of cap-EEG is better spatial spread of electrodes and a larger scalp coverage, which makes it more likely to be sensitive to the source of interest despite varying positions and orientations. Given that the exact source configuration of individual EEG recordings is generally unknown, it is an empirical question whether ear-EEG is sufficient to capture a particular feature of interest of brain activity or not. In the following we will review several studies in which we have used the cEEGrid.

## cEEGrid sensitivity—P300

In Debener et al. (2015) we used a two-tone auditory oddball paradigm to show that a clear P300 ERP can be captured with the cEEGrid. Participants performed the experiment twice in a morning and an afternoon session, approximately 7 h apart. The cEEGrids were fitted in the morning and left in place for the afternoon session, without manipulating the cEEGrids in between. The results revealed reliable P300 effects and reasonable signal quality even in the afternoon session, after wearing the cEEGrids for several hours. Stimulus presentation and EEG acquisition were done with an off-the-shelf Android smartphone. A single-trial analysis resulted in classification accuracies that are similar to those found for cap-EEG data using the same paradigm in stationary (Halder et al., 2010) and mobile conditions (Debener et al., 2012). In summary, this study is in line with previous ones reporting the suitability of ear-EEG for capturing P300 effects (Looney et al., 2012; Bleichner et al., 2015). Note, however, that all these studies were conducted in seated, stationary conditions. While encouraging, it remains to be shown that ear-EEG captures P300 ERPs in truly mobile recording conditions.

## cEEGrid sensitivity—alpha oscillations

**Figure 4:**
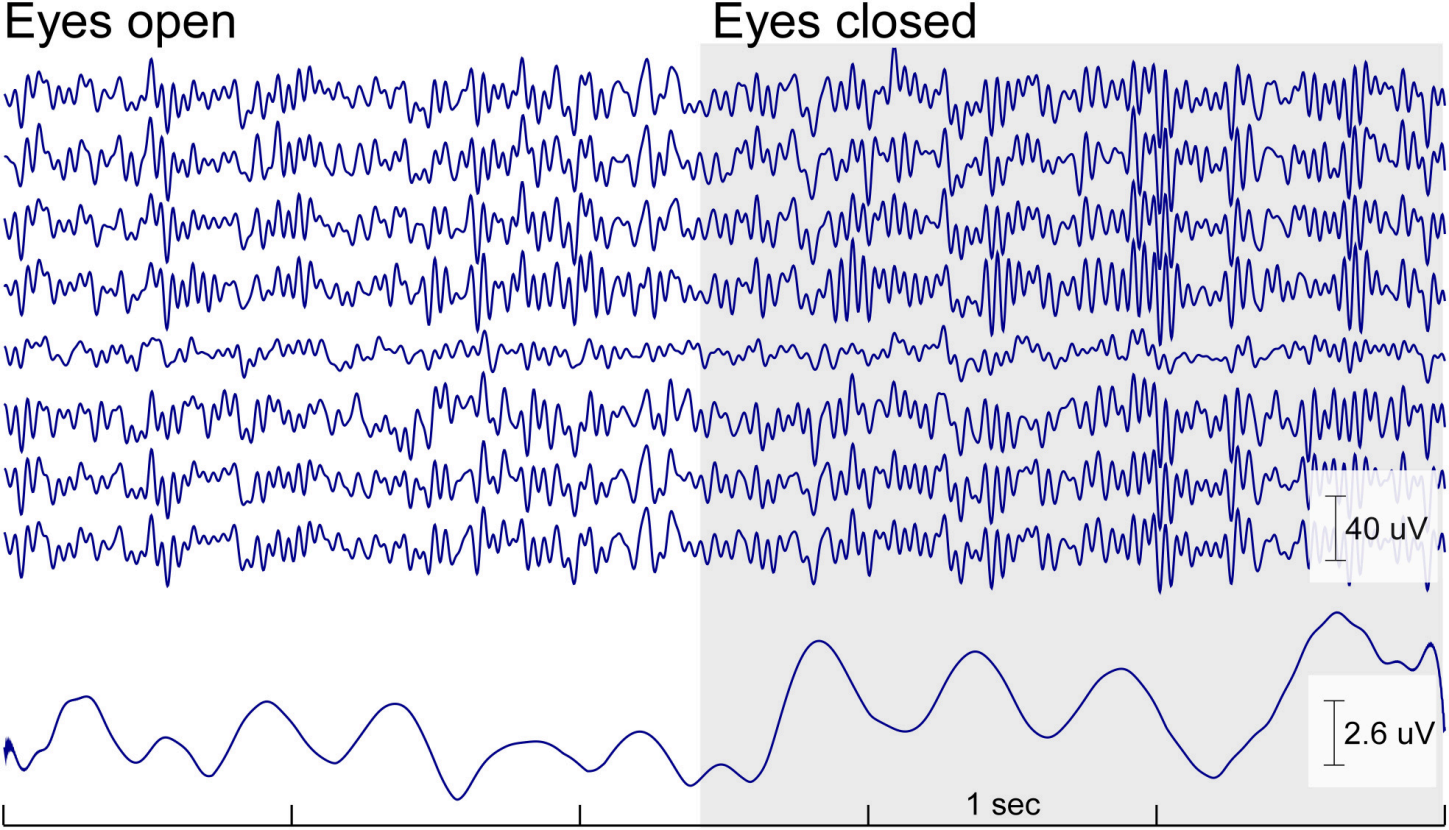
**Continuous cEEGrid EEG data (5 s) with eyes open and eyes closed of one participant for a cEEGrid on the right side**. Below is the mean amplitude of the alpha band (8–12 Hz). The increase in alpha band activity is readily visible in the raw signal and in the amplitude of the alpha band activity as soon as the eyes are closed. The effect is wide spread and can be seen on all cEEGrid channels.

In the same study we asked participants to sit relaxed in their chair with either open or closed eyes. For the morning and the afternoon session a clear increase in alpha power in the eyes closed condition compared to the eyes open condition was observed as expected. Figure 4 shows the raw EEG traces of the cEEGrid at the transition of open to closed eyes. The increase in alpha oscillations is readily apparent.

## cEEGrid sensitivity—comparison to cap-EEG

In two other studies cEEGrid ear-EEG data were directly compared to concurrently recorded high-density cap-EEG data. In these studies participants were equipped with two cEEGrids connected to a mobile amplifier (Smarting; www.mbraintrain.com) and a high-density EEG cap connected to a standard lab amplifier (BrainAmp; www.brainproducts.com). The EEG was collected from both setups concurrently and time-synchronized using the lab streaming layer software framework (Kothe, 2014). Mirkovic et al. (2016) found that the continuous EEG, as recorded with the cEEGrid, can be used to identify the attended speaker in a double speaker paradigm (Mirkovic et al., 2015; O'Sullivan et al., 2015). In this paradigm, the participants attend to one of two simultaneously presented speech streams. Based on 1 min chunks of data it was possible to correctly decode to which of the two speakers the participant attended to for 69% of the segments. While this result was significantly worse than the one found for 84-channel cap-EEG data (accuracy 85%), the cEEGrids results are still above statistical chance-level in most participants. Further analyses revealed that the lower classification performance of the cEEGrid was due to the worse spatial coverage, and not strongly affected by the lower channel count. Apparently a complex source configuration contributes to decoding performance, requiring a broad spatial coverage.

**Figure 5:**
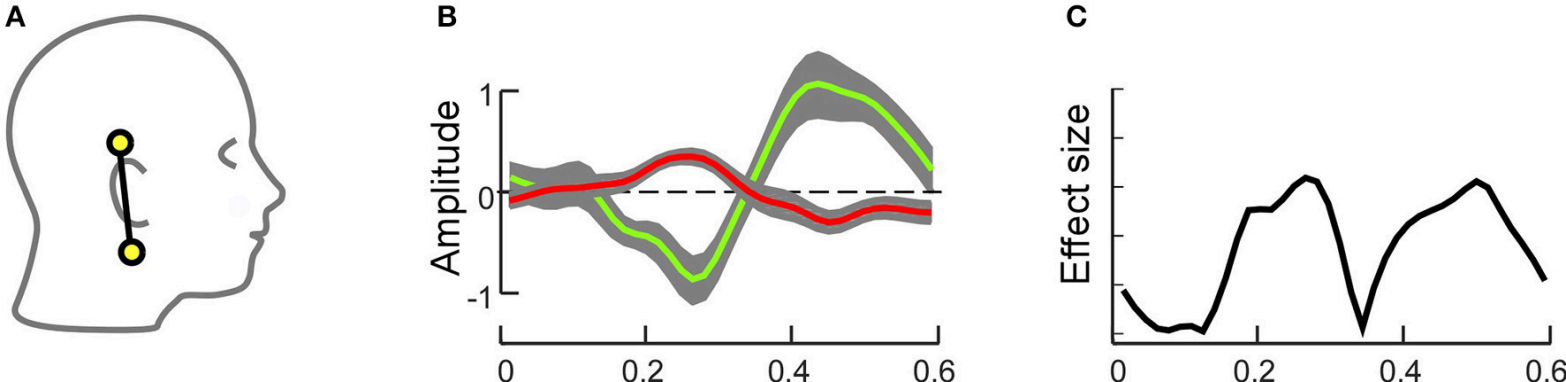
**Grand average single channel result for an auditory attention paradigm as described in Bleichner et al. (**2016**)**. **(A)** cEEGrid channel pair that was used to compute data show in **(B,C)**. **(B)** The grand average ERPs for attended tones (green) and unattended tones (red) showed clear differences in amplitude. Shaded gray areas indicate the standard error of the mean. **(C)** The effect size over time is given as Hedges' g (absolute) values.

In the second study, cEEGrids and high-density cap-EEG data were compared in a spatial auditory attention paradigm (Bleichner et al., 2016). In this study, the participants had to attend to one of three simultaneously presented sound streams. The 3 s long sound streams originated from center, left and right locations and differed in a number of sound features as well as in the number of tones presented (3, 4, and 5, respectively). In this study, a replication of earlier multi-channel cap-EEG results (Choi et al., 2013) was possible with both high-density cap-EEG as well as cEEGrid ear-EEG. For both setups clear attention modulated ERPs in response to the attended sound stream were detected (Figure 5), and comparable single-trial classification accuracies at around 70% were achieved. Classification performance was not significantly different between cap-EEG and ear-EEG, which further suggests that the source configuration driving the condition effect in this paradigm was captured well enough with ear-EEG, resulting in no clear loss of information when compared to cap-EEG.

In the following section, we present evidence from unpublished pilot recordings, to illustrate potential applications (use cases) for cEEGrid-based transparent EEG systems.

## Sleep staging

Conventional EEG is widely used in sleep laboratories, for both clinical and research purposes. Sleep may be seen as a brain wide phenomenon and different sleep stages are characterized by different and possibly distributed generators (Murphy et al., 2009; Dehghani et al., 2010). Traditional sleep EEG recordings use bipolar derivations between a mastoid and a central location (e.g., C4, Campbell, 2009). Recently, Zibrandtsen et al. (2016) reported a first case study of sleep staging using ear-EEG and compared ear-EEG and cap-EEG. Although the amplitudes of the ear-EEG recordings were considerably smaller when compared to those derived from cap-EEG channels, ear-EEG may allow for an identification of sleep stages (see also Stochholm et al., 2016). Compared to cap-EEG, ear-EEG has the advantage of a high wearing comfort, which should give rise to better sleep EEG recording quality. cEEGrids adhere firmly to the skin and should therefore be suitable for overnight recordings. To explore this idea, we conducted several sleep-EEG recordings on ourselves, with cEEGrids attached to the wireless Smarting amplifier.

**Figure 6:**
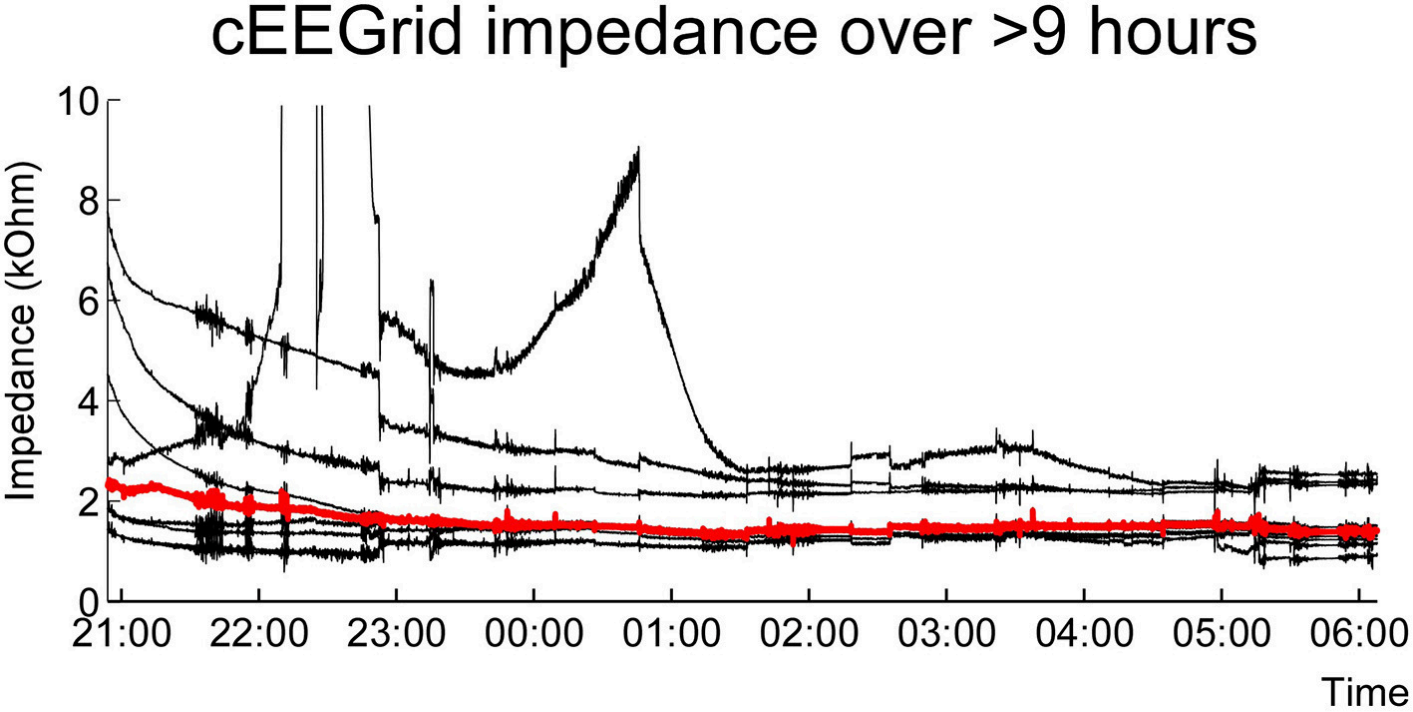
**Single subject sleep EEG recording over the course of a night**. Shown are the electrode impedance values recorded in parallel with EEG. Indicated in red is the median impedance score for all channels.

**Figure 7:**
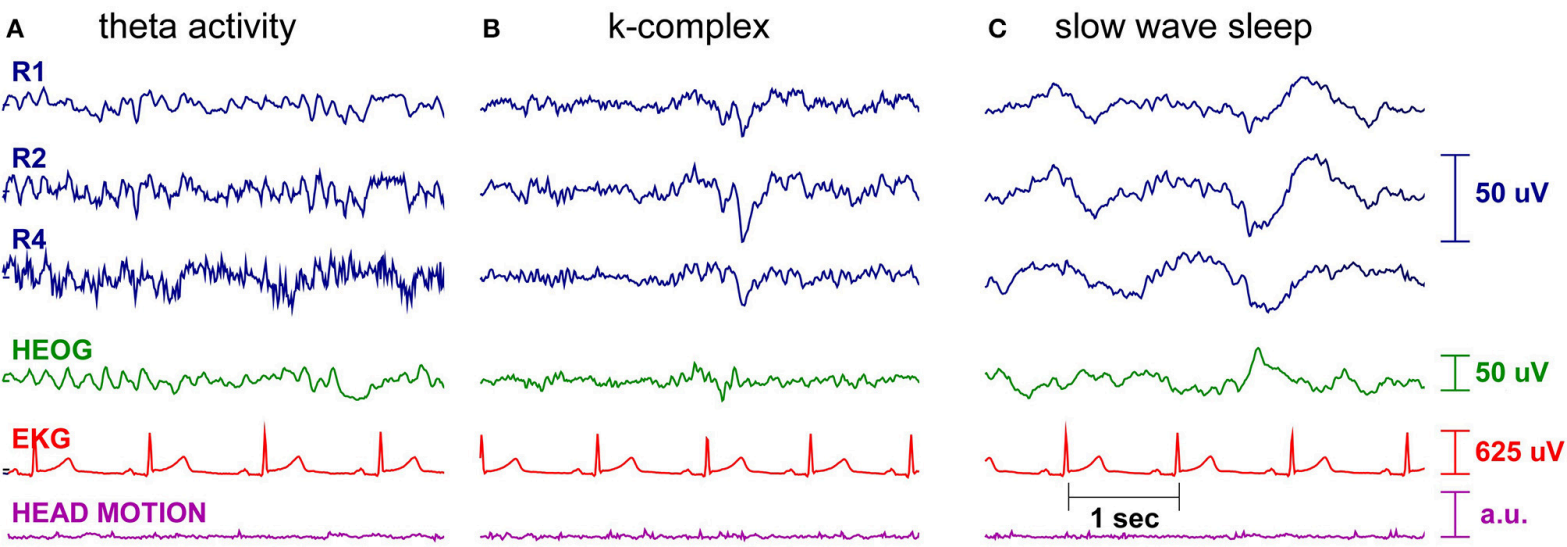
**Single subject sleep EEG recording**. Shown are three right ear cEEGrid EEG channels (blue), the horizontal electrooculogram (green), the electrocardioagram (red), and the head motion, as measured by the Smarting amplifier gyroscope (purple). Different characteristic sleep patterns are clearly visible, such as **(A)** theta activity, **(B)** k-complexes, and **(C)** slow wave sleep.

Figures 6, 7 show cEEGrid data recorded from one of the authors (SD) over a period of 9 h. One cEEGrid was attached to the right ear and the amplifier was attached to the neck with medical tape. Wireless data acquisition was performed with a smartphone placed at bedside. The Smarting amplifier allows for continuous monitoring of channel voltage and impedance data. This enabled us to monitor channel impedances over night. As can be seen in Figure 6, impedances dropped initially and then remained fairly constant throughout the night (with one exception at approximately 10 p.m., which was caused by physical interference of the cEEGrids with frame sides (from a pair of glasses). As illustrated in Figure 7, different EEG sleep features comprising theta activity, k-complexes and slow wave sleep could be captured with the cEEGrid. Compared to in-ear EEG (Zibrandtsen et al., 2016) the cEEGrid ear-EEG amplitudes are somewhat larger.

## Epilepsy EEG

We argue that a clear advantage of the cEEGrids is their high wearing comfort. At present this interpretation is based on qualitative evaluation, a proper multi-subject study comparing wearing comfort of EEG caps versus cEEGrids from our group is currently in preparation. In many patients suffering from epilepsy long-term EEG evaluation is needed to guide therapeutic interventions, and these patients typically report great discomfort. Some may even experience pain when using EEG electrodes glued to their head for days. Epilepsy has a high prevalence in the general public and can have grave effects on the individual. The etiology of mesial temporal lobe epilepsy for example is still not well understood and often refractory to antiepileptic drugs (Téllez-Zenteno and Hernández-Ronquillo, 2012). Long term EEG monitoring could help to develop a better understanding of the individual problems (Curia et al., 2014), as it would increase, for instance, the likelihood of capturing seizures. Long term, ambulatory ear-centered EEG could also help in getting a better picture of the temporal characteristics of the epileptiform activity over extended periods of time (Do Valle et al., 2014). Finally, long term EEG could be used for epilepsy monitoring on a daily basis and could be part of a closed-loop system e.g., for seizure warning.

Common to all these applications is the need to continuously monitor EEG activity. With a transparent EEG this can be achieved in an inconspicuous and socially acceptable manner without the risk of stigmatization for the patient or otherwise limiting him/her in everyday activities. For instance, children could attend school which they usually avoid due to the cosmetic impact of cap-EEG systems (Do Valle et al., 2014). Given the high prevalence of epilepsy ear-EEG could be beneficial for a large number of people.

**Figure 8:**
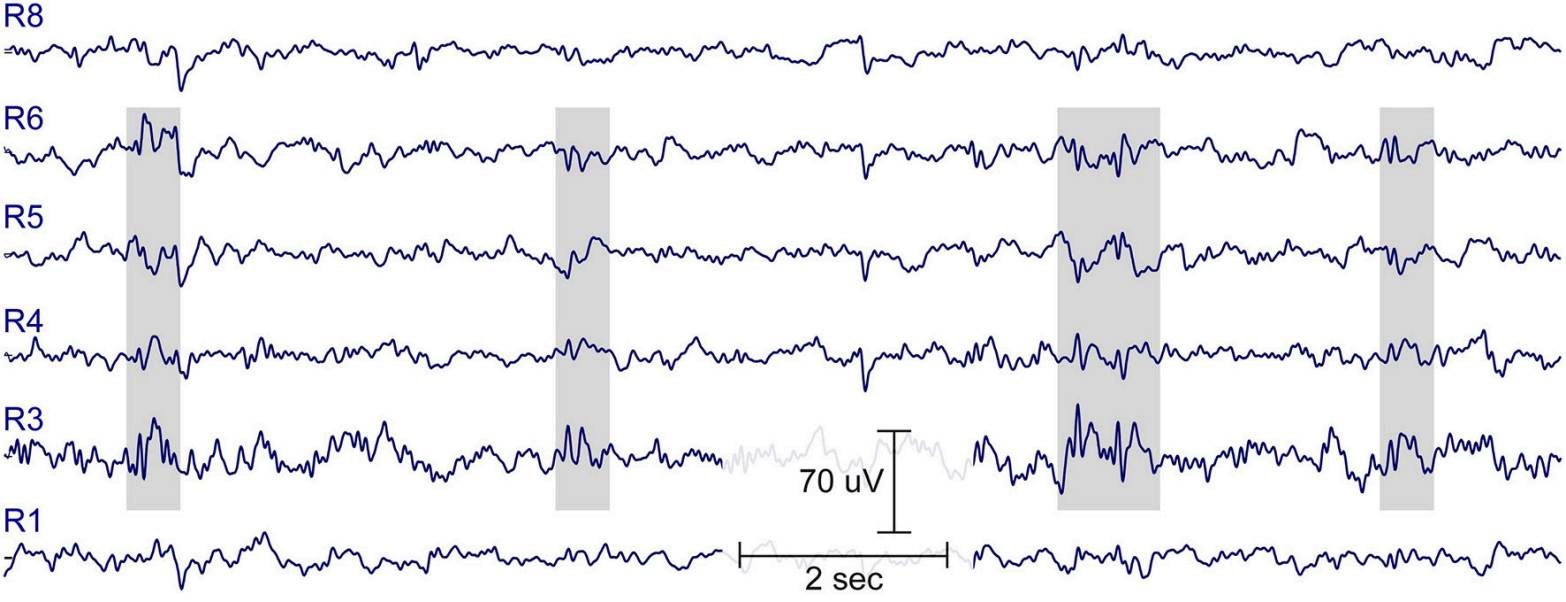
**Fifteen-second resting EEG data from a healthy boy, 7-years of age, with no history of epileptic seizures, whose older brother is diagnosed with rolando epilepsy**. Note spike-wave EEG activity, indicated in gray. See Figure 2 for illustration of electrode labels on cEEGrid.

Figure 8 shows an illustrative example of epileptiform brain activity from one child (boy, 7 years). Two cEEGrids were attached around the ears and connected to the amplifier (SMARTING). Data was acquired for a few minutes, and spike-wave activity was evident by visual inspection. Long-term ear-EEG cEEGrid studies are needed to evaluate whether cEEGrids improve wearing comfort, while preserving the clinical value of the standard cap-EEG recordings.

## Non-neural physiological signals

EEG is intended to record cerebral activity, but the recorded signal may safely be assumed to always consist of a mixture of neural and non-neural contributions. An unknown number of neural sources may originate from cortical and subcortical regions and project differently to the scalp based on source depth and orientation (see above). Non-neural contributions can be subdivided into non-physiological (e.g., thermal noise, power line interference) and physiological signals. The latter may originate from movements of the tongue, the eyes (eye blinks, lateral and vertical eye movements), heart-electrical activity, respiration and other muscle related activity patterns. These signals may carry important information and can be used in their own right (see Wascher et al., 2014, for an example of an eye-blink informed EEG analysis).

**Figure 9:**
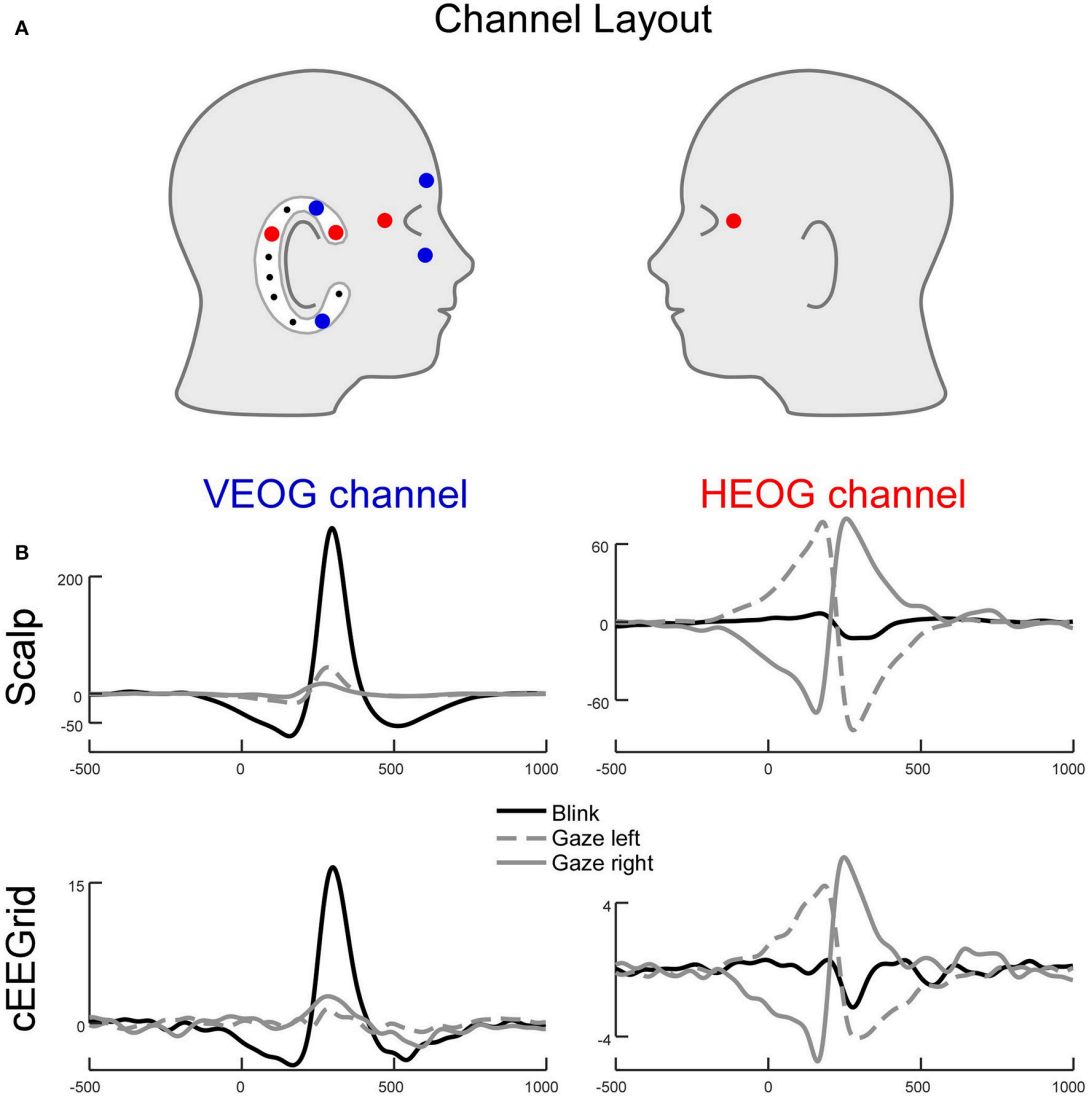
**Lateral eye movements and eye blinks recorded simultaneously with cEEGrid and conventional electrooculogram (EOG) channels. (A)** Channel layout for EOG and selected cEEGrid channels. The VEOG was the bipolar derivation between an above and a below eye electrode. The cVEOG was the bipolar derivation between the cEEGrid channels R2 and R7. The HEOG was the bipolar derivation between a channel next to the left and right eye. The cHEOG was the bipolar derivation between the cEEGrid channels R1 and R4. **(B)** Eye blinks are clearly reflected in a vertically oriented cEEGrid channel, whereas lateral eye movements are clearly reflected in a horizontally oriented cEEGrid channel. Note the similarity in morphology and latency between EOG and cEEGrid channels.

Eye and eyelid movements are among the strongest signals present in EEG recordings (Plöchl et al., 2012) and can be observed at various scalp locations. Consequently, eye movement related potential changes are also present in the cEEGrid data. Figure 9 shows an illustrative example of how eye movement activity is represented in cEEGrid recordings. One of the authors (MGB) performed guided eye movements with leftward and rightward eye movements as well as eye blinks. We recorded the eye movements with a traditional vertical (VEOG) and a horizontal (HEOG) bipolar EOG channel with electrodes directly attached around the eyes. For the cEEGrid, we computed a horizontal bipolar channel between an electrode behind and in front of the ear (R1 minus R4, cHEOG), which corresponds to a traditional HEOG channel in its orientation, and a bipolar channel between an electrode above and below the ear (R2 minus R7, cVEOG) that corresponds to a traditional VEOG channel in its orientation (Figure 9, top). As can be seen, the HEOG and the cHEOG channels were both sensitive to horizontal eye movements but not to eye blinks, whereas the VEOG and the cVEOG channels were sensitive to eye blinks but not lateral eye movements (Figure 9, bottom). From the time course of the (c)VEOG channel, the direction of the vertical eye movement could be determined. This single-subject example indicates, that eye movements are picked up by the cEEGrid and the example further underlines the principle of differential sensitivity of bipolar cEEGrid channels. Furthermore, it exemplifies that eye artifact processing for cEEGrid ear-EEG recordings is advisable.

## Heart rate

**Figure 10:**
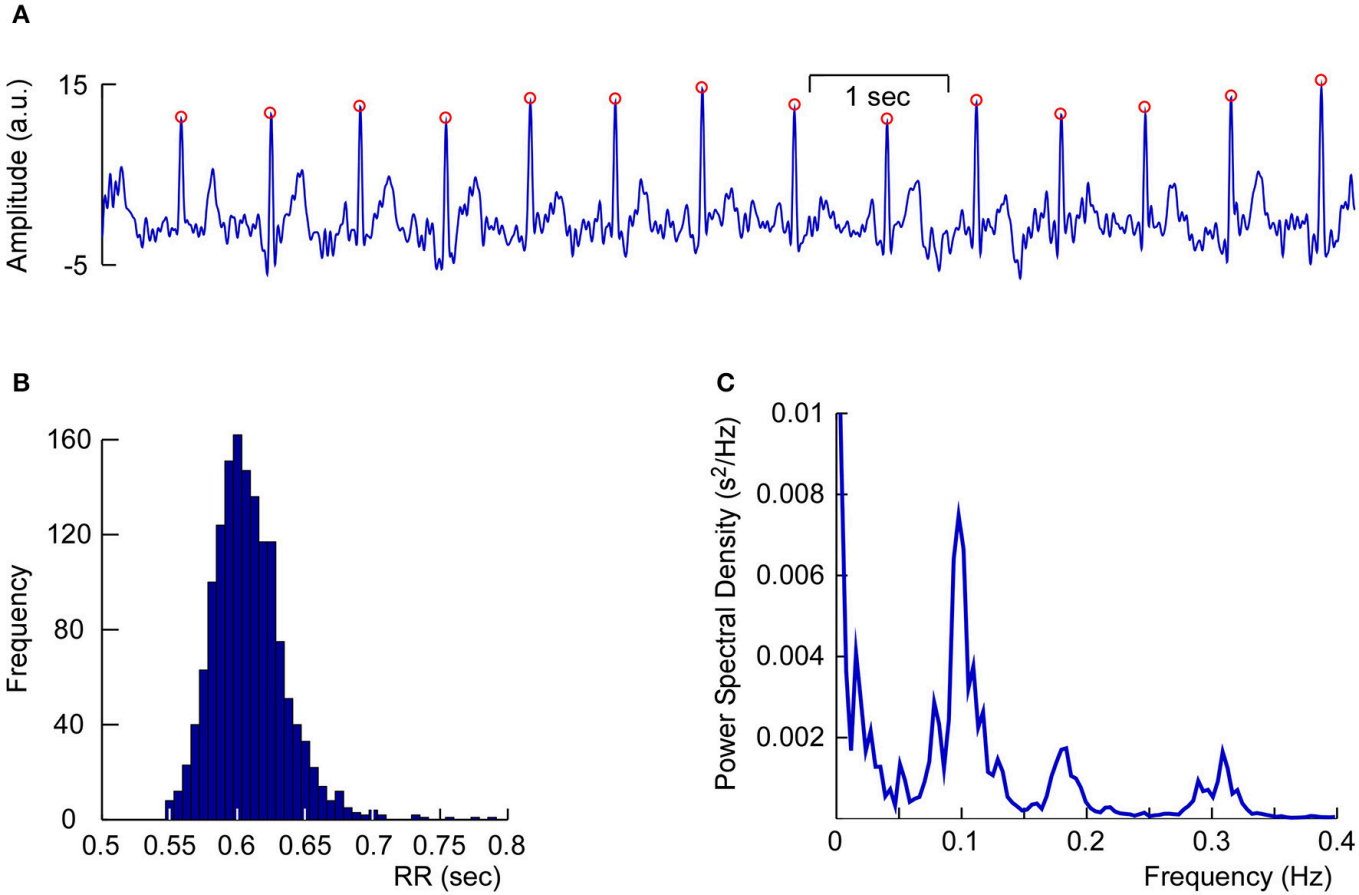
**Single-subject Heart-rate variability analysis based on multi-channel cEEGrid recordings. (A)** Linear decomposition of multi-channel cEEGrid signals with independent component analysis revealed one independent component representing heart-electrical activity, as indicated by prominent R-peaks (red circles). **(B)** Histogram of the RR interval, further supporting reliable R-peak detection. **(C)** Power spectrum analysis of inter-beat intervals, showing typical spectral signatures, such as 0.10 Hz activity.

Another non-neural component that is often found in cap-EEG recordings is the heart beat artifact. Heart beat artifacts in EEG and MEG recordings can be readily identified by the regular periodic time course and the characteristic shape of the QRS complex. Generally this is considered an artifact and removed from the ongoing EEG (Jung et al., 2000; Campos Viola et al., 2009; Onton and Makeig, 2009) but in some situations the simultaneous recording of EEG and electrocardiogram (EKG) is beneficial (Terhaar et al., 2012). Figure 10 shows an exemplary dataset of one of the author's (SD) cEEGrid-EKG, which was found after linear decomposition of the cEEGrid signals with independent component analysis (ICA; Campos Viola et al., 2009; Terhaar et al., 2012). Clearly visible is the QRS complex and the pronounced R wave with its regular pattern, which allowed us to analyze heart-rate variability based on the cEEGrid-EKG. In our experience, ICA recovers the EKG from cEEGrid recordings in approximately 50–60% of all datasets. This is similar to cap-EEG, where ICA identified EKG signals only in a subsample of all datasets (Campos Viola et al., 2009). The presence or the absence of the EKG in cap-EEG and ear-EEG recordings may be due to individual differences in how strongly heart-electrical activity volume conducts to the head.

## Movement artifact

A third class of artifact arises from movement, and traditionally any kind of gross movement is avoided in EEG recordings as movement maybe detrimental to the EEG signal quality. Besides electromyogram (EMG) artifacts caused by muscle activity, movement artifacts in EEG may originate either from the movement of the electrodes relative to the skin or movements of the cables (Ödman and Åke Öberg, 1982; Simakov and Webster, 2010; Cömert and Hyttinen, 2015).

The cEEGrid has some design properties that in principle should reduce the level of movement artifacts. First, the electrode and cable positions are fixed in the cEEGrid, which means that they can hardly move relative to each other. Second, the electrodes are firmly attached to the skin with adhesive tape. Therefore, there is hardly any movement of the electrodes relative to the skin, or of the conduction gel relative to the skin or the electrodes.

## Outlook

We have discussed here what is currently possible with ear-centered cEEGrid EEG acquisition and provide some preliminary evidence supporting its potential for future applications. By supporting and complementing previous pioneering work (Looney et al., 2012), it is our experience that ear-EEG can capture brain signals, in some cases equally well as classical cap-EEG. We expect an increasing number of studies conducted in different laboratories to present further converging evidence on the applicability and usefulness of the ear-EEG concept. In our view, ear-EEG is the only feasible solution toward future transparent EEG technology. We predict that, once fully transparent EEG becomes available, cognitive-controlled hearing aids, epilepsy seizure warning devices, neurofeedback home training systems, sleep staging trackers, workload-adaptive learning programs, and real-time vigilance monitoring/alerting solutions will (continue to) mature into useful, assistive technology. However, as outlined below, a rather large number of problems have to be solved before EEG signals can be used reliably as the key source of information for these purposes.

Currently only one ear-EEG solution is commercially available to the research community (www.ceegrid.com). It may be best described as a research platform, which can help to evaluate whether ear-EEG signals provide sufficient sensitivity and specificity to support a particular application. For instance, it may soon be possible to control hearing aid settings by using neural features derived from real-time cap-EEG signals as acquired in the lab, but whether a similar system performance resulting in higher hearing-aid user satisfaction can be obtained with transparent EEG technology used in daily-life settings remains to be shown. Stepping out of the lab and into the “wild,” uncontrolled ambulatory acquisition is far from trivial and may be regarded as a challenge even bigger than developing classical EEG technology into transparent EEG systems. However, it is obvious that (near) transparent EEG technology is required to make the step from the lab into real-life settings. Therefore, solving technological problems appears to have priority at present.

Our vision of transparent EEG is a device that can be used on a daily basis over extended periods of time without interfering with the user's normal behavior. To enable this it has to be small and portable, motion-tolerant and self-applicable. Furthermore, it needs to be unobtrusive, nearly invisible and comfortable to wear. Note that a complete transparent EEG system consists not only of miniature electrodes; it also requires a miniature amplifier wirelessly sending the data to a mobile acquisition unit such as a smartphone. Ideally, this acquisition unit has signal processing and feedback capabilities to support BCI applications.

High portability implies that all components of a transparent EEG device are small, light-weight and power-efficient. Moreover, they have to be robust against physical strain and water resistant to some degree, so that they can be used for extended periods of time. Sensors such as the cEEGrid and modern smartphones come close to fulfilling these requirements, and recent developments in amplifier technology are also promising (e.g., Zhou et al., 2016) and show that transparent EEG is technically within reach. In our view, it would be desirable to integrate EEG amplifier technology into hearing aids. A close integration of both would enable signal amplification close to sensor sites in a system sufficiently compact to be placed behind the ear.

Achieving motion tolerance is certainly the key toward EEG acquisition in real-life settings. We see clear benefits of such developments not only for applied research, but also for increasing the ecological validity of fundamental research programs. Note that physiological artifacts caused by electrical activity of the muscles are unavoidable, but may be relatively easy to deal with by temporal filter application and other post-processing procedures (Reis et al., 2014). Motion of sensors, and motion of parts of the measurement system, however, may contribute strong interference and corrupt signals to an extend that the underlying brain activity cannot be recovered (Kline et al., 2015). New hardware solutions that account for sensor motion (Yazicioglu et al., 2010; Reis et al., 2014; Cömert and Hyttinen, 2015; Goverdovsky et al., 2015) and miniaturized amplifier solutions featuring shielding and active amplification (Metting van Rijn et al., 1990) may help to alleviate this problem further. In our experience, light-weight head-mounted systems that wirelessly transmit amplified EEG signals may give rise to reasonable motion-tolerance (Debener et al., 2012). Future EEG technology should facilitate the integration of EEG and motion sensor signals to address this problem. For instance, a gyroscope/accelerometer unit placed on the head (e.g., as part of an ear-EEG amplifier) could support a real-time pedometer, and give rise to the real-time attenuation of gait cycle related motion artifacts.

Self-applicability and near invisibility are necessary for easy and frequent EEG use. At first glance dry EEG sensors may be a solution. Yet, dry electrodes as they are most commonly used for classical scalp EEG (Lopez-Gordo et al., 2014) require a net, cap or another supporting structure to keep them in place, are relatively bulky, may cause comfort problems when used over many hours, and the high impedance skin-electrode contact is detrimental for systems aiming for good motion tolerance (Bertrand et al., 2013; Tautan et al., 2014). The cEEGrid solution in its present state is imperfect as well in these regards. It is too large to be near invisible. We envision a solution that consists of a disposable, skin-colored, adhesive sensor array with pre-gelled miniature sensors printed on a flexible and stretch-tolerant silicone layer. Mass-production of such a sensor array should enable marketing the device at low cost, and a well defined reliable connector solution should facilitate use with different amplifiers. Recent EEG sensor developments have shown the general feasibility of soft and flexible electrodes (Norton et al., 2015). For long-term recordings, sensor arrays should either stay in place or be easily removed and reapplied without skin irritations.

A transparent EEG device needs to be simple and robust enough so that the user can use it without risking to damage the system and worry about signal quality. Modern amplifiers feature online impedance measurement, which allows for online signal quality checks. Besides, high usability requires user-friendly software solutions and easy hardware integration and communication. These latter requirements are common to the increasing family of wearable and tracking devices, and we therefore believe that learning from current wearable solutions will help to develop transparent EEG systems.

cEEGrids provide more spatial information than in-ear EEG systems, and it is our experience that this helps to capture and identify features of interest. We consider the cEEGrid as one step toward fully transparent EEG. However, many more steps, including, but not limited to the ones outlined in this report, have to be taken before transparent EEG systems mature into truly assistive, everyday life technology.

## Conclusion

We have introduced the transparent EEG concept and summarized the current state of development of the cEEGrid technology. cEEGrids, in combination with wireless, smartphone-based EEG acquisition and stimulus presentation, represent our first steps toward a truly transparent EEG. It should be emphasized that the current system does not fulfill all criteria for a transparent EEG as defined in the introduction, further developments are necessary. However, the anecdotal evidence presented here illustrates the potential of cEEGrid ear-EEG technology for applications such as sleep and epilepsy, beyond the already validated auditory attention and speech monitoring applications. We hope that this article encourages the further development of next-generation, transparent EEG technology—to achieve ecologically valid, unobtrusive brain activity monitoring.

## Ethics statement

All recordings with the cEEGrid were carried out in the accordance with the recommendations of the local ethics committee (“Kommission für Forschungsfolgenabschätzung und Ethik”; University Oldenburg) with written informed consent from all participants in accordance with the Declaration of Helsinki.

## Author contributions

MB and SD contributed in equal parts to the manuscript regarding conceptualisation, data acquisition, analysis, and writing.

### Conflict of interest statement

The cEEGrid (Debener et al., 2015) concept was developed in our lab based on our previous experience with ear centered EEG and miniature electrodes (Bleichner et al., 2015). Funding for the first version of the cEEGrid, which was produced by TMSI (Oldenzaal, The Netherlands), came from Task Group 7 of the Cluster of Excellence Hearing4All, Oldenburg, Germany. We could show that the cEEGrid allows us to reliably record EEG signals and convinced TMSI to continue the development and market cEEGrids. The authors do not have any financial relationship with TMSI and the authors do not profit from cEEGrid sales financially. Our aim is to develop ear-EEG solutions and make them available to the research community (www.ceegrid.com).
